# Between Delusions and Borders: Diagnosing Delusional Disorder in Migratory Contexts

**DOI:** 10.1192/j.eurpsy.2024.1276

**Published:** 2024-08-27

**Authors:** L. M. Carrasqueira, M. D. Pereira, I. Marques, M. Silva, C. Laureano

**Affiliations:** ^1^Psychiatry and Mental Health Department, Hospital Centre of Leiria, Leiria; ^2^Child and adolescent psychiatry, Coimbra Hospital and University Centre, Coimbra, Portugal

## Abstract

**Introduction:**

The mental health of immigrants is a significant, yet often overlooked, aspect of public health. This case study highlights the intersection of migration and mental health, focusing on a patient with delusional disorder. It is particularly relevant for psychiatrists due to the unique challenges in diagnosing and treating mental health conditions in migrant populations, who often face cultural, linguistic, and systemic barriers in accessing care.

**Objectives:**

The primary objective of this case study is to elucidate the diagnostic and clinical challenges encountered in managing delusional disorder in a migrant patient. The case study presented aims to provide insights into how delusional beliefs can precipitate and perpetuate the process of migration.

**Methods:**

The case study was developed through comprehensive psychiatric interviews during the patient’s stay in a Psychiatric Inpatient Unit, supplemented by a targeted literature review on PubMed using “delusion disorder” and “immigration” as keywords.

**Results:**

The patient, a 44-year-old Indian male, was a functional young adult until 2007 when he began exhibiting symptoms of delusional disorder. His delusions progressively evolved from local scenarios to national and eventually to a global scale. The initial delusions were focused on personal and professional conspiracies within his home country, leading to his first internal migration. As the condition worsened, his delusions expanded, fueling a belief in a widespread conspiracy that transcended national borders. This escalation of delusional beliefs became the primary motivation for the patient’s international migration. He changed countries four times, each move driven by an attempt to escape the perceived threats and conspiracies associated with his delusional disorder. The patient’s journey through various countries was a direct result of the intensifying nature of his condition.

**Conclusions:**

This case study accentuates the profound impact that a delusional disorder can have as a driver and catalyst of international migration, influencing the individual’s decision-making process and shaping the migratory experiences. It emphasizes the necessity for psychiatrists to consider the unique socio-cultural contexts of migrant patients in diagnosis and treatment. The case study advocates for a comprehensive treatment approach, integrating psychiatric care with a nuanced understanding of the migrant’s experiences and challenges. This multifaceted approach is crucial in addressing the complex needs of patients with delusional disorder in migrant populations.

**Disclosure of Interest:**

None Declared

